# Infant Nutritional Status and Markers of Environmental Enteric Dysfunction are Associated with Midchildhood Anthropometry and Blood Pressure in Tanzania

**DOI:** 10.1016/j.jpeds.2017.04.005

**Published:** 2017-08

**Authors:** Lindsey M. Locks, Ramadhani S. Mwiru, Expeditho Mtisi, Karim P. Manji, Christine M. McDonald, Enju Liu, Roland Kupka, Rodrick Kisenge, Said Aboud, Kerri Gosselin, Matthew Gillman, Andrew T. Gewirtz, Wafaie W. Fawzi, Christopher P. Duggan

**Affiliations:** 1 Department of Nutrition, Harvard T.H. Chan School of Public Health, Boston, MA; 2 Management and Development for Health, Dar es Salaam, Tanzania; 3 African Academy for Public Health, Dar es Salaam, Tanzania; 4 Departments of Pediatrics and Child Health, Muhimbili University of Health and Allied Sciences, Dar es Salaam, Tanzania; 5 Division of Gastroenterology, Hepatology and Nutrition, Boston Children's Hospital, Boston, MA; 6 Clinical Research Center, Boston Children's Hospital, Boston, MA; 7 UNICEF Headquarters, New York, NY; 8 Department of Microbiology and Immunology, Muhimbili University of Health Sciences, Dar es Salaam, Tanzania; 9 Department of Population Medicine, Harvard Medical School, Boston, MA; 10 Institute of Biomedical Sciences, Georgia State University, Atlanta, GA; 11 Department of Epidemiology, Harvard T.H. Chan School of Public Health, Boston, MA; 12 Department of Global Health and Population, Harvard T.H. Chan School of Public Health, Boston, MA

**Keywords:** child health, growth, nutrition transition, BMIZ, Body mass index-for-age z-score, EED, Environmental enteric dysfunction, HAZ, Height-for-age z-score, LAZ, Length-for-age z-score, LPS, Lipopolysaccharide, WAZ, Weight-for-age z-score, WHO, World Health Organization, WLZ, Weight-for-length z-score

## Abstract

**Objective:**

To assess whether growth and biomarkers of environmental enteric dysfunction in infancy are related to health outcomes in midchildhood in Tanzania.

**Study design:**

Children who participated in 2 randomized trials of micronutrient supplements in infancy were followed up in midchildhood (4.6-9.8 years of age). Anthropometry was measured at age 6 and 52 weeks in both trials, and blood samples were available from children at 6 weeks and 6 months from 1 trial. Linear regression was used for height-for-age z-score, body mass index-for-age z-score, and weight for age z-score, and blood pressure analyses; log-binomial models were used to estimate risk of overweight, obesity, and stunting in midchildhood.

**Results:**

One hundred thirteen children were followed-up. Length-for-age z-score at 6 weeks and delta length-for-age z-score from 6 to 52 weeks were associated independently and positively with height-for-age z-score and inversely associated with stunting in midchildhood. Delta weight-for-length and weight-for-age z-score were also positively associated with midchildhood height-for-age z-score. The 6-week and delta weight-for-length z-scores were associated independently and positively with midchildhood body mass index-for-age z-score and overweight, as was the 6-week and delta weight-for-age z-score. Delta length-for-age z-score was also associated with an increased risk of overweight in midchildhood. Body mass index-for-age z-score in midchildhood was associated positively with systolic blood pressure. Serum anti-flagellin IgA concentration at 6 weeks was also associated with increased blood pressure in midchildhood.

**Conclusions:**

Anthropometry at 6 weeks and growth in infancy independently predict size in midchildhood, while anti-flagellin IgA, a biomarker of environmental enteric dysfunction, in early infancy is associated with increased blood pressure in midchildhood. Interventions in early life should focus on optimizing linear growth while minimizing excess weight gain and environmental enteric dysfunction.

**Trial registration:**

ClinicalTrials.gov: NCT00197730 and NCT00421668.

The prevalence of childhood overweight and obesity is increasing in almost every region of the world.^1^ At the same time, several low- and middle-income countries continue to confront high rates of undernutrition and impaired child growth, and are thus simultaneously facing a dual burden of malnutrition among children. In low-resource settings in particular, where 156 million children under 5 years of age experience chronic undernutrition,^2^ the widespread prevalence of undernutrition and impaired growth in early life may have profound impacts on long-term health outcomes. Children who suffer from undernutrition in early life are more likely to have reduced adult stature, impaired cognitive development, lower earning potential, and if they are women, also a higher risk of birth complications.^3^ Furthermore, the developmental origins of health and disease paradigm proposes that perturbations in the homeostasis of a developing fetus or infant result in long-term changes affecting that individual's risk of future diseases.^4^ Several studies have documented an increased risk of cardiometabolic risk factors, such as high blood pressure, dyslipidemia, and insulin resistance, among individuals who were born at a low birth weight^5^, ^6^, ^7^, ^8^ and among those with a low body mass index-for-age z-score (BMIZ) in early childhood.^6^, ^9^, ^10^ The relative importance of perinatal versus infant growth in long-term health outcomes, however, is unknown. Furthermore, much of the evidence on the relationship between early life growth and adult health outcomes comes from wealthy nations,^5^, ^9^, ^11^ despite the growing evidence that the relative influence on growth in early life on later-life health outcomes may be population specific.^12^ Specifically, there is limited research from sub-Saharan Africa, where the risk factors for poor growth as well as chronic disease outcomes are likely quite different from those in developed nations.

Recent data suggest that environmental enteric dysfunction (EED), a subclinical condition of the small intestine characterized by villous atrophy, crypt hyperplasia, increased intestinal permeability, inflammatory cell infiltrate, and malabsorption, is associated with growth failure and stunting.^13^, ^14^, ^15^ Some have also hypothesized that EED may increase the risk for cardiometabolic diseases in later life, including insulin resistance and hypertension.^16^ Although the gold standard for assessing EED is small bowel biopsy, the invasive and expensive nature of these procedures has led researchers to pursue biomarkers that are more suitable for widespread use in community-based settings. Our group has assessed previously the use of anti-flagellin and anti-lipopolysaccharide (LPS) IgA and IgG as biomarkers of EED. We found that anti-LPS and anti-flagellin IgA and IgG concentrations increased over the first year of life in Tanzanian infants, that the concentrations in Tanzanian infants were significantly higher than in healthy controls in Boston, and that elevated anti-LPS and anti-flagellin IgA and IgG concentrations were associated with an increased risk of underweight in infancy.^17^ In our current study, we assess whether these biomarkers continue to be associated with growth and health outcomes in midchildhood.

Given the limited research on how nutritional status and EED in early infancy relate to long-term growth and cardiometabolic risk factors in children in low-resource settings, we collected data from a cohort of children in Dar es Salaam, Tanzania, to assess the relative importance of infant growth and nutritional status at age 6 weeks of age and change from 6 to 52 weeks of age, as well as biomarkers of EED in infancy, on health and growth outcomes in midchildhood.

## Methods

The study sample included children born in Dar es Salaam, Tanzania, who participated in 1 of 2 randomized controlled trials of multiple micronutrient supplementation to infants. The first trial (ClinicalTrials.gov: NCT00197730) randomized 2387 infants born to HIV-infected mothers to either daily administration of multiple micronutrients (vitamins B complex, C, and E) or placebo at 6 weeks of age.^18^ Randomization of infants occurred between August 2004 and November 2007; follow-up ended in May 2008. The micronutrient supplements did not show an effect on mortality, morbidity, or child growth.^18^, ^19^ The second trial (ClinicalTrials.gov: NCT00421668) was implemented with a 2 × 2 factorial design assessing the effect of zinc, zinc plus multivitamins (the same combination of vitamins B complex, C, and E as described), multivitamins alone, or placebo among 2400 infants born to HIV-negative women.^20^ The second trial found that zinc supplementation reduced the risk of acute respiratory and diarrheal infections,^20^ but that neither supplement alone nor in combination had an effect on rates of stunting, wasting, or underweight.^21^ The 2 studies were designed to allow for pooled analyses—they were conducted in overlapping clinics with similar staff, they used identical inclusion/exclusion criteria (other than maternal HIV status), and they collected the same sociodemographic and clinical data on all mothers and children. In both trials, infants were randomized at 6 weeks of age, and mothers were asked to bring the children to the clinic for follow-up visits every 4 weeks after randomization. At each monthly follow-up visit, a trained study nurse measured child anthropometry using standard techniques.^22^ Weight was measured on a digital infant balance scale with 10-g precision (Tanita, Arlington Heights, Illinois) and length with 1-mm precision using a rigid length board with an adjustable foot piece.

For the current study, we identified children who participated in the 2 original trials who met the following criteria: children with complete physical descriptions of their home addresses on file, who had anthropometric data at 6 weeks, who had participated in their original trial through 15 months of age, and who were available for contact during the follow-up study recruitment period of June to August 2014. From the 2387 children in the first trial, a list of all children who fulfilled these criteria was generated and simple random sampling was used to select children for follow-up. From the 2400 children in the second trial, we selected from 269 children who had participated in an enteric disease substudy because these children had provided blood specimens at both 6 weeks and 6 months of age.^17^ Further inclusion criteria in the substudy was that children had length-for-age z-score (LAZ) > −2 at 6 weeks.

### Laboratory Assessments

Microtiter plates were coated with purified *Escherichia coli* flagellin (100 ng/well) or purified *Escherichia coli* LPS (2 mg/well). Serum samples from study participants were diluted 1:200 and applied to wells coated with flagellin or LPS. After incubation and washing, the wells were incubated with anti-human IgA (KPL, Milford, Massachusetts) or IgG (GE Healthcare, Little Chalfont, United Kingdom) coupled to a horseradish peroxidase. The quantification of total immunoglobulins was performed with the use of the colorimetric peroxidase substrate tetramethylbenzidine, and absorbance (optical density) was read at 450 nm with the use of an enzyme-linked immunosorbent assay plate reader. Data are reported as optical density-corrected data by subtracting background concentrations, which were determined from the readings in samples that lacked serum.

### Follow-Up Assessments

For all children who participated in the follow-up study, trained study nurses measured their weight, height, and blood pressure when the children were between 4 and 9 years of age. Diastolic, systolic, and mean arterial blood pressure in mm Hg were measured with a DINAMAP DPC120X-EN (GE Medical Systems Information Technologies Inc, Milwaukee, Wisconsin). Blood pressure measurements were assessed 5 times by a single observer and then averaged. Body weight was measured with an electronic digital scale accurate to 0.1 kg (Tanita), and standing height was measured with a stadiometer to the nearest 0.1 cm.

Ethical approval for both parent trials was granted by the Harvard T.H. Chan School of Public Health Human Subjects Committee, the Muhimbili University of Health and Allied Sciences Committee of Research and Publications, the Tanzanian Institute for Medical Research, and the Tanzanian Food and Drug Authority; the follow-up study was approved by the Harvard T.H. Chan School of Public Health Human Subjects Committee and the Muhimbili University of Health and Allied Sciences Committee of Research and Publications. All mothers provided written informed consent to enroll themselves and their children in the original and follow-up studies.

### Statistical Analyses

Details on data collection and management from the 2 trials have been published previously.^18^, ^19^, ^20^, ^21^ In brief, data were double entered by using Microsoft Access software and converted to SAS datasets (version 9.4; SAS Institute, Cary, North Carolina) for analysis. For growth analyses, we calculated age- and sex-specific z-scores for 3 anthropometric indices during infancy: weight-for-length z-score (WLZ), LAZ, and weight-for-age z-score (WAZ) using the 2006 World Health Organization (WHO) growth standards.^23^ In accordance with WHO recommendations, we set all extreme LAZ (<−6 or >6), WLZ (<−5 or >5), and WAZ (<−6 or >5) values to missing.^24^ For follow-up anthropometric data, we calculated age- and sex-standardized height-for-age z-score (HAZ), WAZ, and BMIZ using the 2006 WHO growth standards for children younger than 5 years of age and the WHO 2007 Growth Reference for children aged 5 years and older.^25^ In accordance with WHO guidelines, stunting was defined as HAZ <−2, and overweight as BMIZ >1, and obese as BMIZ >2.

Descriptive statistics, including means with standard deviations and frequencies with percentages, were used to summarize sociodemographic information on maternal, child, and household characteristics as well as the growth characteristics of children who participated in the follow-up study. Our outcomes of interest were HAZ, BMIZ, and WAZ; systolic and diastolic blood pressures; and overweight, obesity, and stunting in midchildhood. Our exposures of interest were baseline anthropometric indicators (6-week LAZ, WAZ, and WLZ) and change in each anthropometric indicator from 6 to 52 weeks of age, as well as EED biomarkers (anti-flagellin and anti-LPS IgG and IgA) at 6 weeks and 6 months of age. For each continuous outcome of interest, we first conducted univariate linear regression models with each anthropometric and EED biomarker as predictors of interest. Confounders for multivariate models were selected based on a review of the literature and included which trial the child originally participated in, the trial treatment arm, and the child's sex for all models; maternal height and maternal education for all models for anthropometric outcomes; and child's gestational age at birth as well as age at midchildhood follow-up for all blood pressure models. Multivariate models assessing the effect of anthropometry in infancy included each anthropometric indicator at 6 weeks of age as well as the change in the same indicator from 6 to 52 weeks of age to assess relative importance of the 2 time periods for each indicator. The assumption of linearity was assessed using plots of residuals versus predicted values, and normality based on normal quantile plots.

For our primary outcomes (anthropometry and blood pressure in midchildhood), data from 113 subjects and 8 covariates in the multivariate linear regression models, provided 80% power to detect a minimal increase of 7% in the R^2^ value for a predictor of interest at significance level of 0.05. In models where biomarkers of EED were predictors of interest, the number of subjects was reduced to 66 in our analysis, which provided 80% power to detect a minimal increase of 12% in R^2^ value at a significance level of 0.05. We also conducted univariate and multivariate log-binomial regression models to assess the relationship between each of our predictors of interest and overweight, obesity, and stunting in midchildhood. Given the limited power to assess these outcomes, multivariate models for binary anthropometric outcomes only adjust for which trial the child participated in, the child's sex, and the corresponding anthropometric indicator at baseline or change from 6 to 52 weeks of age. Multivariate models for stunting in midchildhood also adjust for maternal height. All analyses were conducted in SAS version 9.4 (SAS Institute).

## Results

Research staff attempted to contact 327 children and successfully reconsented and re-enrolled 113 children, 47 from the first trial (children born to HIV-infected mothers, none of whom had tested positive for HIV) and 66 from the second trial (children born to HIV-uninfected mothers). All 66 children from the second trial had blood samples drawn at 6 weeks and 6 months of age. There were no differences in sociodemographic characteristics or early infant growth indicators when comparing children who participated in the follow-up study and the original trials with the exceptional of gestational age in weeks, which was slightly longer in children in the follow-up study compared with the parent trials (40.0 ± 2.3 vs 39.4 ± 2.7). Mean LAZ, WLZ, and WAZ were already below 0 at 6 weeks of age, and the means continued to decline through age 12 months (Table I). The age range for children at follow-up was 4.6-9.8 years (6.8 ± 1.6 years). Children born to HIV-infected mothers (participants in trial 1) were older at follow-up than children born to HIV-uninfected mothers (participants in trial 2). The prevalence of stunting, thinness, overweight, and obesity at follow-up were 4.4%, 5.3%, 8.0%, and 3.5%, respectively.

**Table I:** Characteristics of the 113 children who participated in midchildhood (4-9 years of age) assessments

Maternal characteristics	
Age (y)	28.1 ± 5.0*
Height (cm)	157.2 ± 6.0
HIV infected, n (%)	47 (41.6)
Prior pregnancies, n (%)	
0	31 (27.9)
1-4	74 (66.7)
≥5	6 (5.4)
Years of formal education	7.8 ± 2.7
Employment, n (%)	
Housewife without income	61 (55.0)
Housewife with income	39 (35.1)
Other	11 (9.9)
Household characteristics	
Household possessions†, n (%)	
0	31 (27.9)
1-2	31 (27.9)
≥3	49 (44.1)
Child characteristics at baseline (5-7 wk)	
Age at randomization (wk)	5.8 ± 0.3
Male sex, n (%)	51 (45.1)
Birthweight (kg)	3.16 ± 0.46
Gestational age at birth (wk)	40.0 ± 2.3
LAZ	−0.18 ± 1.03
WAZ	−0.27 ± 0.88
WLZ	−0.12 ± 1.10
Flagellin IgG (adjusted optical density)‡	0.43 ± 0.18
Flagellin IgA (adjusted optical density)‡	0.30 ± 0.23
LPS IgG (adjusted optical density)‡	0.72 ± 0.38
LPS IgA (adjusted optical density)‡	0.41 ± 0.31
Child characteristics during 12-month follow-up in original trials	
Change in LAZ from baseline to 12 months	−0.52 ± 1.08
Change in WAZ from baseline to 12 months	−0.35 ± 1.06
Change in WLZ from baseline to 12 months	−0.49 ± 1.52
Child characteristics at follow-up in midchildhood (4-9 y)	
Age (y)	6.8 ± 1.6
HAZ	−0.47 ± 1.1
WAZ	−0.54 ± 1.0
BMIZ	−0.40 ± 1.0
Stunting (HAZ <−2)	5 (4.4%)
Overweight (BMIZ >1)	9 (8.0%)
Obesity (BMIZ >2)	4 (3.5%)
Thinness (BMIZ <−2)	6 (5.3%)
Systolic blood pressure (mm Hg)	91.8 ± 9.9
Diastolic blood pressure (mm Hg)	54.1 ± 6.5
Mean arterial blood pressure (mm Hg)	64.8 ± 7.7

* Mean ± SD for all continuous variables; number and frequency (%) for all categorical variables.

† From a list that includes sofa, television, refrigerator and fan.

‡ Only available in children in trial 2 (n = 66).

In multivariate models predicting HAZ in midchildhood, LAZ at age 6 weeks and change in LAZ, WLZ, and WAZ from 6 to 52 weeks of age were all correlated positively with HAZ in midchildhood (Table II). Of note, in the model containing 6-week LAZ and change in LAZ from 6 to 52 weeks of age, both variables were significant, independent predictors of midchildhood HAZ (Beta 0.47 [95% CI 0.25-0.68; *P* < .001] and 0.40 [95% CI 0.20-0.61; *P* < .001], respectively). None of the EED biomarkers at 6 weeks or 6 months of age were associated significantly with HAZ or any of the anthropometric indicators in midchildhood. We also did not find that treatment in either trial was associated with HAZ in midchildhood or any of the anthropometric indicators.

**Table II:** Linear regression models for HAZ, BMIZ, and WAZ in midchildhood (4-9 years of age)

	HAZ		BMIZ		WAZ	
	Univariate models	Multivariate models*	Univariate models	Multivariate models*	Univariate models	Multivariate models*
Baseline (6-week) measurements						
LAZ†						
Beta	0.33	0.47	0.04	0.16	0.25	0.42
95% CI	0.14 to 0.53	0.25 to 0.68	0.14 to 0.23	0.06 to 0.39	0.06 to 0.43	0.22 to 0.62
*P* value	.001	<.001	.65	.15	.009	<.001
WAZ†						
Beta	0.06	0.12	0.35	0.53	0.28	0.46
95% CI	0.19 to 0.30	0.13 to 0.37	0.14 to 0.56	0.30 to 0.76	0.06 to 0.50	0.24 to 0.67
*P* value	.65	.33	.002	<.001	.01	<.001
WLZ†						
Beta	−0.34	−0.05	0.28	0.64	−0.02	0.43
95% CI	0.52 to −0.16	0.29 to 0.19	0.11 to 0.45	0.42 to 0.87	−0.20 to 0.16	0.21 to 0.65
*P* value	<.001	.11	.001	<.001	.82	<.001
Flagellin IgG (adjusted optical density)‡						
Beta	−1.32	−1.36	0.36	0.46	−0.57	−0.52
95% CI	−3.12 to 0.48	−3.11 to 0.38	−1.15 to 1.87	−1.10 to 2.02	−2.15 to 1.00	−2.09 to 1.04
*P* value	.15	.12	.64	.56	.47	.50
Flagellin IgA (adjusted optical density)‡						
Beta	−0.02	−0.82	0.24	−0.15	0.19	−0.58
95% CI	−1.44 to 1.40	−2.26 to 0.62	−0.93 to 1.42	−1.42 to 1.13	−1.04 to 1.41	−1.86 to 0.69
*P* value	.98	.26	.68	.82	.76	.36
LPS IgG (adjusted optical density)‡						
Beta	0.01	−0.44	−0.11	−0.19	−0.08	−0.40
95% CI	−0.84 to 0.86	−1.28 to 0.41	−0.81 to 0.60	−0.94 to 0.55	−0.81 to 0.66	−1.14 to 0.34
*P* value	.99	.31	.77	.60	.84	.28
LPS IgA (adjusted optical density)‡						
Beta	−0.61	−0.93	−0.06	−0.08	−0.41	−0.61
95% CI	−1.62 to 0.41	−1.91 to 0.05	−0.91 to 0.79	−0.97 to 0.80	−1.29 to 0.47	−1.48 to 0.27
*P* value	.24	.06	.90	.86	.35	.17
Change in anthropometry from 6 to 52 weeks						
LAZ†						
Beta	0.19	0.40	0.13	0.19	0.21	0.40
95% CI	−0.01 to 0.38	0.20 to 0.61	−0.05 to 0.30	−0.02 to 0.40	0.03 to 0.39	0.21 to 0.59
*P* value	.06	<.001	.16	.08	.02	<.001
WAZ†						
Beta	0.28	0.35	0.21	0.32	0.33	0.45
95% CI	0.09 to 0.48	0.14 to 0.56	0.03 to 0.39	0.13 to 0.51	0.16 to 0.51	0.27 to 0.63
*P* value	.004	.001	.02	<.001	<.001	<.001
WLZ†						
Beta	0.33	0.29	0.07	0.38	0.27	0.45
95% CI	0.21 to 0.46	0.11 to 0.47	−0.06 to 0.20	0.21 to 0.54	0.15 to 0.38	0.29 to 0.62
*P* value	<.001	.002	.28	<.001	<.001	<.001

* Multivariate models for HAZ, BMIZ, and WAZ in mid-childhood adjust for which trial the child originally participated in, treatment arm, maternal height (cm), maternal education (0, 1-7 or ≥8 years), and child's sex and age at follow-up. Multivariate models for BMIZ adjust for which trial the child originally participated in, treatment arm, maternal height (cm), maternal education (0, 1-7 or ≥8 years), and child's sex and age at follow-up. Multivariate models for WAZ adjust for which trial the child originally participated in, treatment arm, maternal height (cm), maternal education (categories), and child's sex and age at follow-up.

† Multivariate models with anthropometric indicators as independent variables also include the corresponding anthropometric indicator at 6 weeks or change from 6 to 52 weeks of age, for example, the model reporting the beta parameter for LAZ at 6 weeks adjusts for change in LAZ from 6 to 52 weeks of age, and vice versa.

‡ n = 66 (children from second trial only).

In the multivariate model adjusting for sociodemographic characteristics, WLZ at 6 weeks of age and change in WLZ from 6 to 52 weeks of age were associated independently with BMIZ in midchildhood (Beta 0.64 [95% CI 0.42-0.87; *P* < .001] and 0.38 [95% CI 0.21-0.54; *P* < .001], respectively). Similarly, WAZ at 6 weeks of age and change from 6 to 52 weeks of age were also associated independently with an increase in BMIZ in midchildhood, although the effect estimates were slightly smaller. In multivariate models, 6-week LAZ was not associated significantly with BMIZ in midchildhood, whereas the change in LAZ from 6 to 52 weeks of age was marginally, significantly correlated with BMIZ at follow-up. In multivariate models for WAZ in midchildhood, baseline LAZ and change in LAZ from 6 to 52 weeks of age were correlated independently positively with WAZ, as were baseline WAZ and change in WAZ, and baseline WLZ and change in WLZ.

None of the anthropometric indicators at baseline, nor changes from 6 to 52 weeks of age, were significant predictors of systolic or diastolic blood pressures at follow-up in the univariate or multivariate models (Table III). Each additional unit in BMIZ in midchildhood, however, was associated with an increase in systolic blood pressure of 2.21  mm Hg (95% CI 0.40-4.02; *P* = .02). We also found that flagellin IgA at 6 weeks was a significant predictor of systolic blood pressure in midchildhood in models adjusted for sociodemographic characteristics (Figure). Flagellin IgA at 6 weeks of age was also associated linearly with diastolic blood pressure in midchildhood in multivariate models. To enhance interpretability, we compared quartiles of flagellin IgA and found that children in the highest quartile of flagellin IgA at 6 weeks of age had a mean systolic blood pressure that was 10.3 mm Hg higher (95% CI 2.49-18.11) than infants in the lowest quartile of flagellin IgA at 6 weeks of age in the multivariate models. In additional analyses, we included baseline values and changes in pediatric anthropometry in multivariate models assessing flagellin IgA at 6 weeks of age as a predictor of systolic and diastolic blood pressure in midchildhood; flagellin IgA at 6 weeks of age remained a significant predictor of both blood pressure measures, even after adjusting for infant anthropometry. None of the EED biomarkers at 6 months of age were significant predictors of either blood pressure variable in multivariate models.

**Figure:**
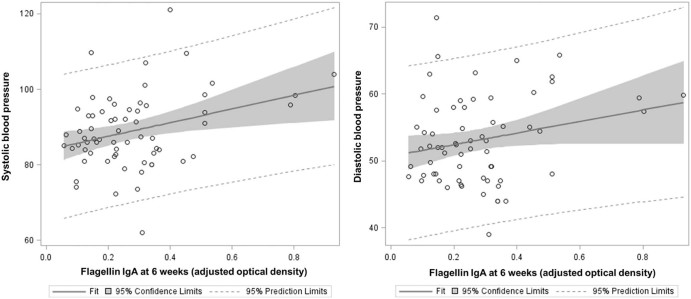
Flagellin IgA at 6 weeks and systolic and diastolic blood pressure in midchildhood (4-9 years of age).

**Table III:** Linear regression models for systolic and diastolic blood pressure in midchildhood (4-9 years of age)

	Systolic blood pressure		Diastolic blood pressure	
	Univariate models	Multivariate models*	Univariate models	Multivariate models*
Baseline (6-week) measurements				
LAZ†				
Beta	0.24	0.44	0.43	0.60
95% CI	−1.57 to 2.05	−1.73 to 2.61	−0.75 to 1.62	−0.84 to 2.05
*P* value	.79	.69	.47	.41
WAZ†				
Beta	0.64	1.52	0.50	0.94
95% CI	−1.50 to 2.79	−0.87 to 3.91	−0.90 to 1.89	−0.65 to 2.52
*P* value	.55	.21	.48	.24
WLZ†				
Beta	0.26	2.04	−0.07	0.85
95% CI	−1.46 to 1.97	−0.44 to 4.52	−1.19 to 1.04	−0.80 to 2.50
*P* value	.77	.11	.90	.31
Flagellin IgG (adjusted optical density)‡				
Beta	−0.97	4.58	3.60	7.36
95% CI	−15.23 to 13.30	−12.11 to 21.27	−6.17 to 13.36	−4.30 to 19.01
*P* value	.89	.58	.46	.21
Flagellin IgA (adjusted optical density)‡				
Beta	17.67	17.81	12.08	13.95
95% CI	7.51 to 27.82	6.50 to 29.1	5.10 to 19.07	6.14 to 21.76
*P* value	<.001	.003	.001	.001
LPS IgG (adjusted optical density)‡				
Beta	−2.23	−0.58	1.19	2.29
95% CI	−8.84 to 4.38	−8.19 to 7.02	−3.36 to 5.74	−3.05 to 7.63
*P* value	.50	.88	.60	.39
LPS IgA (adjusted optical density)‡				
Beta	−2.21	−1.71	−1.34	−1.39
95% CI	−10.19 to 5.78	−10.67 to 7.25	−6.83 to 4.15	−7.72 to 4.94
*P* value	.58	.70	.63	.66
Change in anthropometry from 6 to 52 weeks				
LAZ†				
Beta	0.58	0.80	−0.04	0.40
95% CI	−1.15 to 2.31	−1.41 to 3.01	−1.18 to 1.10	−01.07 to 1.87
*P* value	.50	.47	.95	.59
WAZ†				
Beta	0.62	1.10	0.17	0.55
95% CI	−1.16 to 2.39	−0.92 to 3.13	−0.99 to 1.33	−0.79 to 1.89
*P* value	.49	.28	.78	.24
WLZ†				
Beta	0.39	1.25	0.28	0.70
95% CI	−0.97 to 1.76	−0.48 to 2.97	−0.52 to 1.09	−0.45 to 1.85
*P* value	.57	.16	.49	.23
Anthropometry in midchildhood (4-9 years)				
HAZ				
Beta	−0.21	−0.06	−0.51	−0.39
95% CI	−1.86 to 1.44	−1.84 to 1.71	−1.59 to 0.58	−1.57 to 0.79
*P* value	.80	.94	.36	.51
BMIZ				
Beta	1.54	2.21	0.42	0.85
95% CI	−0.26 to 3.35	0.40 to 4.02	−0.78 to 1.62	−0.39 to 2.08
*P* value	.09	.02	.49	.18

* Multivariate models for systolic and diastolic blood pressure control for which trial the child participated in, treatment arm, child's sex, gestational age at birth, and age at mid-childhood follow-up. Multivariate models diastolic blood pressure adjust for which trial the child originally participated in, whether the child was randomized to multivitamin supplementation, maternal height (cm), child's gestational age at birth, and child's age at follow-up visit.

† Multivariate models with anthropometric indicators as predictors also include the corresponding anthropometric indicator at 6 weeks of age or change from 6 to 52 weeks of age, for example, the model reporting the beta parameter for LAZ at 6 weeks adjusts for change in LAZ from 6 to 52 weeks of age, and vice versa.

‡ n = 66 (children from second trial only).

In multivariate models for binary outcomes in midchildhood, we found that both baseline WLZ and change in WLZ from 6 to 52 weeks of age, as well as baseline WAZ and change in WAZ, were associated independently and positively with the risk of being overweight in midchildhood (Table IV; available at www.jpeds.com). Of note, each unit increase in baseline WLZ was associated with a 2.5-fold increase in risk of being overweight in midchildhood (aRR 2.47; 95% CI 1.04-5.90) after adjusting for change in WLZ from 6 to 52 weeks of age, child's sex, and original trial, whereas each unit increase in WLZ from 6 to 52 weeks of age was associated with an increased risk of 2.45 (95% CI 1.40-4.30) in the same model. Change in LAZ from 6 to 52 weeks of age was also associated with an increased risk of overweight in multivariate models. In multivariate models for obesity in midchildhood, baseline WAZ and change in all 3 anthropometric indicators from 6 to 52 weeks of age were associated significantly with an increased risk of obesity. In multivariate models for stunting in midchildhood, baseline LAZ and change in LAZ from 6 to 52 weeks of age were associated independently and negatively with risk of stunting (aRR 0.23 [95% CI 0.08-0.68; *P* = .008] and 0.31 [95% CI 0.13-0.77; *P* = .01], respectively). Change in WAZ and WLZ from 6 to 52 weeks of age were also associated marginally and significantly with a decreased risk of stunting in midchildhood.

## Discussion

In this longitudinal study of 113 Tanzanian children, we found that LAZ at age 6 weeks of age and change in LAZ in the first year of life were both independent, positive predictors of HAZ and independent negative predictors of stunting in midchildhood. We also found that the 6-week WAZ and WLZ, as well as changes in WAZ and WAZ in the first year of life, were associated independently and positively with BMIZ in midchildhood, and associated negatively with risk of overweight and obesity. Although linear growth in infancy was also associated with BMIZ, overweight, and obesity, this relationship was less robust than that with weight-related variables; similarly, weight-related indicators in infancy were less strongly correlated with height variables in midchildhood. BMIZ in midchildhood was associated in turn significantly and positively with systolic blood pressure, and serum anti-flagellin IgA in early infancy was also a significant predictor of midchildhood blood pressure.

Cohort studies in high-income countries have also found that birthweight and weight gain in early life are positively correlated with BMI, overweight, and obesity later in life^26^, ^27^; however, our study is one of few to longitudinally follow-up with infants from a low-income country over many years,^28^, ^29^, ^30^, ^31^, ^32^ particularly in sub-Saharan Africa.^28^ Our findings show that in the resource-limited environment of Dar es Salaam, where poor sanitation, high burdens of infectious disease, and suboptimal infant feeding practices contribute to high levels of undernutrition in infancy,^18^, ^19^, ^20^, ^21^ weight gain in early life continues to track throughout childhood and ultimately correlates with BMIZ and risk of overweight and obesity in midchildhood.

Stunting in early life increases the risk of impaired cognitive development, adult short stature, lower earning potential, and birth complications in women.^3^ We found that LAZ at 6 weeks of age and change in LAZ from 6 to 52 weeks of age were associated independently with height in midchildhood, and that they were also correlated independently and inversely with stunting in midchildhood. Our findings thus emphasize the importance of nutritional status in both the perinatal and infant periods to improve long-term nutrition and health outcomes in children. Other studies have also found that early life linear growth predicts attained height later in childhood or adulthood^29^, ^30^, ^31^, ^32^, ^33^, ^34^, ^35^; however, less clear in the existing literature is the relationship between early life linear growth and later BMI. A recent study pooling data from 5 cohorts in low-income and middle-income countries found that, although change in height in the first 2 years of life was associated with a minor increase in risk of being overweight in adulthood, increases in weight accounting for height in the first 2 years of life was a much stronger predictor of overweight in adulthood.^28^ Our findings support the hypothesis that early life gains in weight, particularly weight relative to height, has negative consequences for increasing BMI and risk of overweight and obesity. We also found that linear growth in infancy was associated with an increased risk of overweight and obesity in midchildhood, though this association was less robust than that of weight-related indicators and midchildhood BMIZ, overweight, and obesity. Further research on how interventions can promote healthy linear growth without resulting in excess weight gain in early infancy will be essential in addressing the global dual burden of disease.

This study identified a strong relationship between flagellin antibodies in early infancy and blood pressure in midchildhood. The relationship between morbidity, particularly diarrheal disease in infancy and malnutrition has been well-documented^1^, ^36^; however, researchers have only recently begun to explore the important role of EED in malnutrition, highlighting that EED may lead to both micronutrient and macronutrient malabsorption.^37^, ^38^, ^39^ Currently, diagnosing EED remains challenging. Small bowel biopsies have been considered the gold standard for the assessment of the mucosal structure; however, given the invasive and expensive nature of these procedures, research is underway to determine biomarkers that may be more appropriate for widespread use in community-based studies.^40^, ^41^ The bacterial protein flagellin mediates bacteria motility and is present in the gut lumen, even in a healthy state; however, flagellin has very limited access to cross the epithelium and reach the mucosal immune system. However, a decrease in gut barrier function or an increase in levels of bacteria that produce flagellin might activate the adaptive immune responses to these molecules, resulting in the generation of anti-flagellin and anti-LPS antibodies, as has been shown to occur in patients with short bowel syndrome^42^ and Crohn's disease.^43^ Our group has previously shown that anti-flagellin IgA and IgG concentrations increased over the first year of life in Tanzanian infants, that the concentrations in Tanzanian infants were much higher than in healthy controls in Boston, and that elevated anti-flagellin IgA and IgG concentrations were associated with an increased risk of underweight in infancy.^17^ Our current study also highlights that, in addition to its role in malnutrition in infancy, EED, as measured by antibodies to bacterial components, may also play an important role in long-term chronic disease risk,^16^ particularly when one considers the research documenting the likelihood of blood pressure tracking from childhood through adulthood.^44^ Of note, in our sensitivity analyses, we added infant anthropometry to the multivariate models linking 6-week flagellin IgA and midchildhood systolic and diastolic blood pressure, and found that this did not reduce substantially the magnitude of the relationship between flagellin IgA in infancy and midchildhood blood pressure. Our findings, thus, support the hypothesis that EED in infancy may affect long-term cardiometabolic outcomes through pathways external to growth, such systemic inflammation and epigenetic changes in immune function.^16^

Our study has several limitations. We do not have measurements in children between infancy and the midchildhood follow-up, and we only have biomarkers of EED at 6 weeks and 6 months of age, so we are limited in our ability to compare critical periods of growth or to evaluate the potential impact of EED on growth at other ages. It is also worth noting that we only measured a limited set of cardiometabolic risk factors that did not include insulin sensitivity or other vascular outcomes, such as vascular reactivity. Our follow-up study sample may also suffer from selection bias. In particular, the least well-off children, that is, those who became ill, died, or moved since the trial ended, are likely to be excluded from our follow-up sample. However, given that developmental programming is strongest when environmental insults are most severe, if our study had included less healthy children (who likely would have experienced greater growth faltering and EED in infancy), we would have expected to see even stronger associations between infant and midchildhood health indicators. By contrast, our study is also limited by the low prevalence of overweight, obesity, and stunting in this peri-urban African setting and, thus, the limited power for analyses of binary outcomes. Our estimates of overweight and obesity in this sample were, however, comparable with other studies in Tanzanian children.^45^, ^46^ Although overweight and obesity are relatively rare in Tanzanian children, a recent study found that 24.1% and 19.2% of adults in Kinondoni, 1 of 3 municipalities in Dar es Salaam, are overweight and obese, respectively.^47^ We showed that weight gain in early life tracks into midchildhood, and evidence from other contexts indicates that weight gain in midchildhood tracks into adulthood.^48^ As sub-Saharan Africa continues to undergo the nutrition transition, it will be increasingly important to monitor weight gain in infancy and as children age.

Our study, and others, longitudinally tracked pediatric growth from infancy into midchildhood in a sub-Saharan African context.^49^, ^50^ Given the rapid pace of the nutrition transition in sub-Saharan Africa and the dual burden of preventing both pediatric undernutrition but also chronic disease later in life,^1^ our study helps to elucidate the relationship between standard growth indicators in early infancy and health outcomes in later life, as well as the possible role of EED in child health. As the global health community continues to invest in the healthy growth and development of children in sub-Saharan Africa, an expanded emphasis beyond short-term outcomes such a survival and treatment of malnutrition and infectious disease will be essential to promote long-term health and prevent obesity and cardiometabolic diseases.

## Figures and Tables

**Table IV:** Relative risks of overweight, obesity, and stunting in midchildhood (4-9 years)

	Overweight (BMIZ > 1; N = 113, overweight n = 9)		Obesity (BMIZ > 2; N = 113, obese n = 4)		Stunting (HAZ < −2; N = 113, stunted n = 6)	
	Univariate models	Multivariate models*	Univariate models	Multivariate models*	Univariate models	Multivariate models*,†
Baseline (6-week) measurements						
LAZ						
Risk ratio	1.24	1.94	1.16	2.46	0.25	0.23
95% CI	0.69-2.22	0.96-3.93	0.46-2.90	0.74-8.20	0.11-0.56	0.08-0.68
*P* value	.48	.07	.76	.14	<.001	.008
WAZ						
Risk ratio	1.46	2.16	1.25	3.33	0.54	0.26
95% CI	0.70-3.01	1.15-4.05	0.41-3.85	1.41-7.87	0.26-1.12	0.04-1.61
*P* value	.31	.02	.69	.006	.10	.15
WLZ						
Risk ratio	1.10	2.47	1.03	3.85	2.38	0.48
95% CI	0.63-1.92	1.04-5.90	0.43-2.45	0.74-20.17	0.98-5.78	0.11-2.04
*P* value	.74	.04	.95	.11	.06	.32
Change in anthropometry from 6 to 52 weeks						
LAZ						
Risk ratio	1.60	2.37	2.03	4.03	0.68	0.31
95% CI	0.99-2.58	1.21-4.63	1.04-3.96	1.32-12.33	0.34-1.36	0.13-0.77
*P* value	.053	.01	.04	.01	.28	.01
WAZ						
Risk ratio	1.67	3.13	2.24	7.54	0.32	0.22
95% CI	1.18-2.36	1.50-6.52	1.40-3.58	3.12-18.22	0.12-0.86	0.04-1.13
*P* value	.004	.002	<.001	<.001	.02	.07
WLZ						
Risk ratio	1.52	2.45	2.10	4.66	0.40	0.27
95% CI	1.04-2.23	1.40-4.30	1.19-3.70	1.59-13.67	0.23-0.70	0.06-1.20
*P* value	.03	.002	.01	.005	.02	.08

* Multivariate models adjust for which trial the child originally participated in and child's sex as well as the corresponding anthropometric indicator at 6 weeks or change from 6 to 52 weeks of age, for example, the model reporting the beta parameter for LAZ at 6 weeks of age adjusts for change in LAZ from 6 to 52 weeks of age, and vice versa.

† Multivariate model for stunting in midchildhood also adjusts for maternal height (cm).
